# Overexpression of astroglial major histocompatibility complex class I in the medial prefrontal cortex impairs visual discrimination learning in mice

**DOI:** 10.1186/s13041-020-00710-5

**Published:** 2020-12-14

**Authors:** Bolati Wulaer, Kazuhiro Hada, Akira Sobue, Norimichi Itoh, Toshitaka Nabeshima, Taku Nagai, Kiyofumi Yamada

**Affiliations:** 1 Department of Neuropsychopharmacology and Hospital Pharmacy, Nagoya University Graduate School of Medicine, 65 Tsurumai-cho, Showa-ku, Nagoya, 466-8560 Japan; 2 Advanced Diagnostic System Research Laboratory, Fujita Health University Graduate School of Health Sciences, Toyoake, 470-1192 Japan

**Keywords:** MHCI, Astrocyte, Touchscreen, Visual discrimination, Reversal learning, Learning and memory, Cognition, Recognition memory

## Abstract

**Background:**

Immune molecules, such as cytokines, complement, and major histocompatibility complex (MHC) proteins, in the central nervous system are often associated with neuropsychiatric disorders. Neuronal MHC class I (MHCI), such as H-2D, regulate neurite outgrowth, the establishment and function of cortical connections, and activity-dependent refinement in mice. We previously established mice expressing MHCI specifically in astrocytes of the media prefrontal cortex (mPFC) using the adeno-associated virus (AAV) vector under the control of the GfaABC1D promoter. Mice expressing the soluble form of H-2D (sH-2D) in the mPFC (sH-2D-expressing mice) showed abnormal behaviors, including social interaction deficits and cognitive dysfunctions. However, the pathophysiological significance of astroglial MHCI on higher brain functions, such as learning, memory, and behavioral flexibility, remains unclear. Therefore, cognitive function in mice expressing sH-2D in astrocytes of the mPFC was tested using the visual discrimination (VD) task.

**Methods:**

sH-2D-expressing mice were subjected to the VD and reversal learning tasks, and morphological analysis.

**Results:**

In the pretraining, sH-2D-expressing mice required significantly more trials to reach the learning criterion than control mice. The total number of sessions, trials, normal trials, and correction trials to reach the VD criterion were also significantly higher in sH-2D-expressing mice than in control mice. A morphological study showed that dendritic complexity and spine density were significantly reduced in the dorsal striatum of sH-2D-expressing mice.

**Conclusion:**

Collectively, the present results suggest that the overexpression of astroglial MHCI in the mPFC results in impaired VD learning, which may be accompanied by decreased dendritic complexity in the dorsal striatum and mPFC.

## Introduction

The brain is considered to be ‘immuno-privileged’ because of the lack of classical immune molecules in the central nervous system (CNS) [1, 2]. However, current research suggests the presence of communication between the immune and nervous system for brain functions after the discovery of immune molecules, such as cytokines, complement, and major histocompatibility complex (MHC) proteins, in the developing and adult brain [3–5]. Among these immune molecules, recent studies highlighted the roles of MHC class I (MHCI) in the brain. MHCI molecules contain a heavy chain and β2-microglobulin light chain [6, 7]. In the immune system, MHCI presents a short polypeptide of 8–10 amino acids from a cytosolic antigen when a cell is infected with a virus. In the CNS, neuronal MHCI molecules regulate neurite outgrowth, cortical connections, activity-dependent refinement in the visual system, and synaptic plasticity [3–5, 8–10]. Glial MHCI molecules are weakly expressed in normal and healthy brains but are up-regulated under pathological conditions, such as viral infection. A systemic immune stimulation in rodents has been shown to activate astrocytes and microglia in the brain [11, 12] and induce MHCI gene expression in non-neuronal cells [13] and MHCII in microglia [14].

To clarify the pathophysiological role of MHCI expression in astrocytes, we previously established mice expressing MHCI specifically in astrocytes of the medial prefrontal cortex (mPFC) using the AAV vector under the control of the GfaABC1D promoter [15]. Mice expressing the soluble form of H-2D (sH-2D) in the mPFC (sH-2D-expressing mice) showed brain dysfunction manifested by impaired social interactions and object recognition memory, which was accompanied by neuropathological changes, including the activation of microglial cells, decreases in parvalbumin-positive cell numbers, and reductions in dendritic spine density in the mPFC. A treatment with GW4869, an inhibitor of exosome synthesis, ameliorated these behavioral and neuropathological changes in sH-2D-expressing mice, suggesting that the overexpression of MHCI in astrocytes affects microglial proliferation as well as neuronal numbers and spine densities, thereby leading to social and cognitive deficits in mice, possibly via exosomes produced by astrocytes [15].

In the present study, we investigated higher brain functions, such as learning, memory, and behavioral flexibility, in sH-2D-expressing mice using the visual discrimination (VD) task. It should be noted that the validation of the animal model is shown in our previous report [15]. The touchscreen-based VD task provides high translational validity to further evaluate neuronal projections for higher-order brain functions in mice [16–20]. Previous studies indicated that the dorsal striatum is important for VD learning [18, 21]. Instrumental action and outcome behaviors are known to depend on the dorsal striatum and its connections with the mPFC [22, 23]. Therefore, we examined the dendritic and spine morphology of medium spiny neurons in mPFC projection terminals, the dorsomedial striatum (DMS), and dorsolateral striatum (DLS) [24].

## Methods

### Animals

C57BL/6J mice (Japan SLC, Shizuoka, Japan) were housed in groups of 4 per cage and maintained under a standard specific pathogen-free environment with a standard 12-h light/dark cycle (lights on at 9:00) at a constant temperature of 23 ± 1 ºC. Animals were given a 1-week acclimatization period prior to the start of the experiments. They were allowed free access to food and water before the initiation of pretraining for the VD and reversal learning tasks. Only male mice were used in the present study to avoid potential estrus cycle-related performance variability in females [25]. Three separate batches of mice were used in the present study (a total of 53 mice). The sample size for each experiment was determined based on our previous studies with the relevant type of experiment [18, 26]. They were randomly subjected to control or sH-2D viral injection groups as follows: VD and reversal learning (control 4 mice, sH-2D-expressing 4 mice; 2 mice were excluded from the analyses because of natural death [18]); Golgi staining (control 4 mice, sH-2D-expressing 4 mice; [26]). Animals were handled in accordance with the guidelines established by the Institutional Animal Care and Use Committee of Nagoya University, the Guiding Principles for the Care and Use of Laboratory Animals approved by the Japanese Pharmacological Society, and the National Institutes of Health Guide for the Care and Use of Laboratory Animals.

### Plasmid and AAV production

The validation of the animal model is shown in our previous report [15]. To specifically target astroglial MHCI in the mPFC, we used pAAV-2/5 (Cell BioLabs Inc.) under the control of the GfaABC1D promotor. The MHCI/GFP was expressed in glial fibrillary acidic protein (GFAP)-positive astrocytes, but not in neurons or microglia. The transfection efficiencies to GFP+ cells/GFAP+ cells in our experimental conditions were above 70% in each group [15]. We produced the plasmid and pAAV-2/5 as described previously [15]. Briefly, cDNA for mouse MHCI was amplified by polymerase chain reaction (PCR) from a mouse brain cDNA library using specific primers (sH-2D forward primer, ATGAATTCGCCGCCATGGGGGCGATGGC; sH-2D reverse primer, ATGTCGACCCATCTCAGGGTGAGGGGCT), and inserted into a pCRII-blunt TOPO vector (Invitrogen, Carlsbad, CA, USA). cDNA was subcloned into the EcoRI site of the expression vector pCAGGS-HA, which was a gift from Dr. Kozo Kaibuchi. In the AAV vector, pZac2.1 gfaABC1D-EGFP-P2A-sH-2D was generated by replacing EGFP-P2A-sH-2D in tdTomato in pZac2.1-gfaABC1D-tdTomato, which was donated by Dr. Baljit Khakh (Addgene plasmid # 44332). AAV vectors were prepared as described previously [15, 27]. Briefly, plasmids for the AAV vector, pHelper (Cell BioLabs Inc., San Diego, CA, USA), and pAAV-2/5 were transfected into HEK293 cells (Cell BioLabs, Inc.) using Lipofectamine 2000 (Invitrogen). After a 3-day incubation, cells were collected and lysed by freeze and thaw cycles. Cell lysates were incubated with benzonase nuclease (Millipore, Darmstadt, Germany). Cell debris was removed by centrifugation at 10,000×*g* at room temperature for 10 min. Supernatants were used as the primary virus. AAV titers were estimated via a quantitative polymerase chain reaction.

### sH-2D-expressing mouse model

We established the sH-2D-expressing mouse model as described previously (Sobue et al. 2018). Briefly, seven-week-old male mice were anesthetized with tribromoethanol (250 mg/kg, i.p.) and positioned in a stereotaxic frame (David Kopf, Tujunga, CA, USA). AAV gfaABC1D-EGFP-P2A-sH-2D (1 × 10^12^ genome copies/ml) was bilaterally injected into the mPFC (+ 1.5 mm anteroposterior, ± 0.5 mm mediolateral from the bregma, − 2.5 mm dorsoventral from the skull) in a volume of 0.5 µl/site, according to the mouse brain atlas [28]. AAV gfaABC1D-EGFP-P2A was injected as a control group. Three weeks after injections, animals were subjected to behavioral or morphological analyses.

### Touchscreen-based VD and reversal learning tasks

**Fig. 1:**
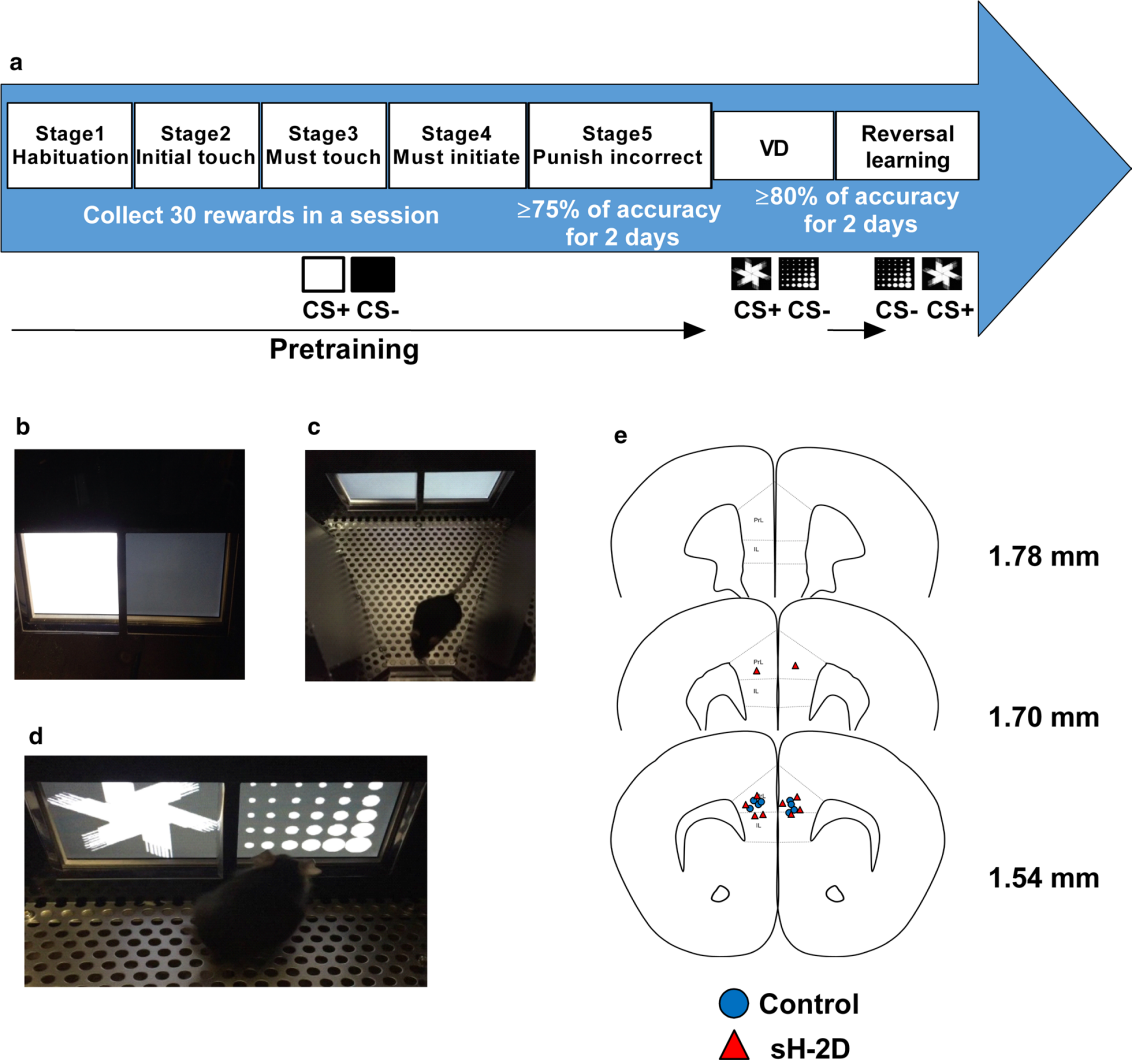
Experimental schedule of pretraining, VD, and reversal learning. **a** Experimental schedule of pretraining, VD, and reversal learning. **b** Stimuli used in pretraining. **c** Mice initiating a new trial by touching the nozzle or receiving a liquid reward through the nozzle. **d** Mice making a decision between a pair of stimuli in VD and reversal learning. **e** The injection sites. CS+, conditioned stimulus associated with a reward; CS-, unconditioned stimulus not associated with a reward; VD, visual discrimination

Tasks were performed using the touchscreen chamber system (Phenosys, Berlin, Germany; Brainscience Idea, Osaka, Japan). The experiments were conducted during the light phase each day (13:00–16:00). The protocol used was described in detail in previous studies [18, 29]. Briefly, access to food and water was restricted for 2 h (17:00–19:00) each day at least 1 week before pretraining in order to provide sufficient motivation to perform the tasks, and food and water restrictions were continued until the end of the task. The body weight was maintained at 85–90% of non-restricted mice. The task started with 5-stage pretraining to shape screen-touch behavior in mice (Fig. 1a). In stage 1, mice were habituated to the touchscreen chamber. They were allowed to freely explore the chamber and rewards were available during the 20-min session. The criterion was to receive 30 rewards (20 μl of milk) on 2 consecutive days. During stage 2, one window of the touchscreen was illuminated with a white plain square for 30 s (Fig. 1b). When the stimulus was offset, the reward nozzle came into the chamber and the reward was delivered. The retrieval of milk initiated an inter-trial interval (ITI) of 20 s before the next image presentation. When the mouse touched the response window during a white plain square presentation, a reward was delivered to accompany the image stimulus termination. Stage 3 proceeded in the same manner as stage 2, except that the mouse was required to touch the response window displaying the image before reward delivery. Each image was displayed until mice touched the response window. The criterion was to receive 30 rewards in a 60-min session at least once. In addition to the stage 3 procedure, mice had to initiate each trial by approaching the nozzle in stage 4 (Fig. 1c). When the trial started, the nozzle was presented in the operant chamber without a reward. Touching the nozzle resulted in the presentation of an image on the touchscreen. The criterion was the same as that for stage 3. In stage 5, mice were introduced to incorrect responses. Mice were punished for touching a blank response window with a 5-s time-out. ITI began after the time-out, and then the next trial was initiated. The criterion in this stage was to complete 30 rewards showing ≥ 75% accuracy in a 30-min session on 2 consecutive days, and mice were then moved to the VD task. To prevent location bias, the stimulus was pseudorandomly presented during all training stages; it never showed more than 3 times on the same side in a row. After mice learned how to operate the touchscreen (> 75% on 2 consecutive days), they were subjected to the VD task. In the VD task, trial initiation was triggered by mice touching the nozzle, and 2 stimuli (marble and fan) were then presented simultaneously in the 2 response windows (Fig. 1d). One of the stimuli was associated with a reward, while the other was not. Stimuli were presented pseudorandomly and not displayed in the same location for more than 3 trials in a row (excluding correction trials). Stimulus contingencies were counterbalanced. Touching the correct response resulted in the delivery of a reward (20 μl of milk). When the incorrect response was touched, the stimuli offset immediately and a 5-s time-out period was started. After ITI (20 s), a correction trial was given instead of a new trial. In the correction trial, the same stimulus set was repeatedly presented in the same location until the mouse made a correct response. The criterion of the task was a more than 80% correct response on 2 consecutive days. The session finished after 60 min or the completion of 30 trials, whichever comes first. The total numbers of trials, correction trials, and correction errors as well as the percentage of correct responses and the perseveration index (the number of correction trials as a ratio of errors) in different training stages were analyzed. The reversal learning task was similar to the initial acquisition of the VD task, except that the contingency of the stimulus pair was reversed. Once a mouse reached the criterion, the contingency of the stimuli was reversed. The previous reward stimulus became an incorrect response, while the previous non-rewarded stimulus became the correct response. The injection sites were checked by immunostaining after the behavioral tasks were completed (Fig. 1e).

### Golgi staining and morphological analyses

Golgi staining was performed using the FD Rapid Golgi Stain Kit according to the manufacturer’s protocol (FD NeuroTechnologies, Ellicott City, MD, USA) and a previous study [15]. Mice were sacrificed 3 weeks after the viral injection without any stimuli. Brains were then sectioned using a cryostat at a thickness of 80 µm. Bright-field microscopic images of neurons located in DMS and DLS were obtained (BZ9000, KEYENCE, Osaka, Japan). Starting from the soma, circles 10 µm apart from each other overlay the dendritic tracings. The length of the dendrites is determined by the number of circles that the dendrites cross over. The intersection is defined as when a branch splits into two sub-branches. Node is the bifurcations on the dendrites and ending is recognized as dendritic endings (terminal ends). Only fully impregnated neurons isolated from neighboring displaying dendritic trees without obvious truncations and impregnated neurons were retained for analyses. We analyzed the secondary or third dendrites on branches of DMS or DLS neurons that were at least 50 μm from the cell body. We measured the spines of 3 dendrites per neuron in 3 neurons per mouse. Structural characteristics of spines are shown in Fig. 5d [27]. All images were traced using Neurolucida software (MicroBrightField Bioscience, Williston, VT, USA) and analyzed by NeuroExplorer (MicroBrightField). These analyses were performed using 12 slices per mouse from 4 mice in each group.

### Data analyses

All data were expressed as means ± SEM. Statistical analyses were performed with GraphPad Prism 6.0 (GraphPad Software, Inc., CA, USA). Differences between two groups were analyzed by a two-tailed Student’s t test. Multiple group comparisons were conducted using the analysis of variance, followed by Tukey’s test. The criterion for a significant difference was **p < 0.01 or *p < 0.05 for all statistical evaluations.

## Results

### Performance of sH-2D-expressing mice in pretraining and VD task

**Fig. 2:**
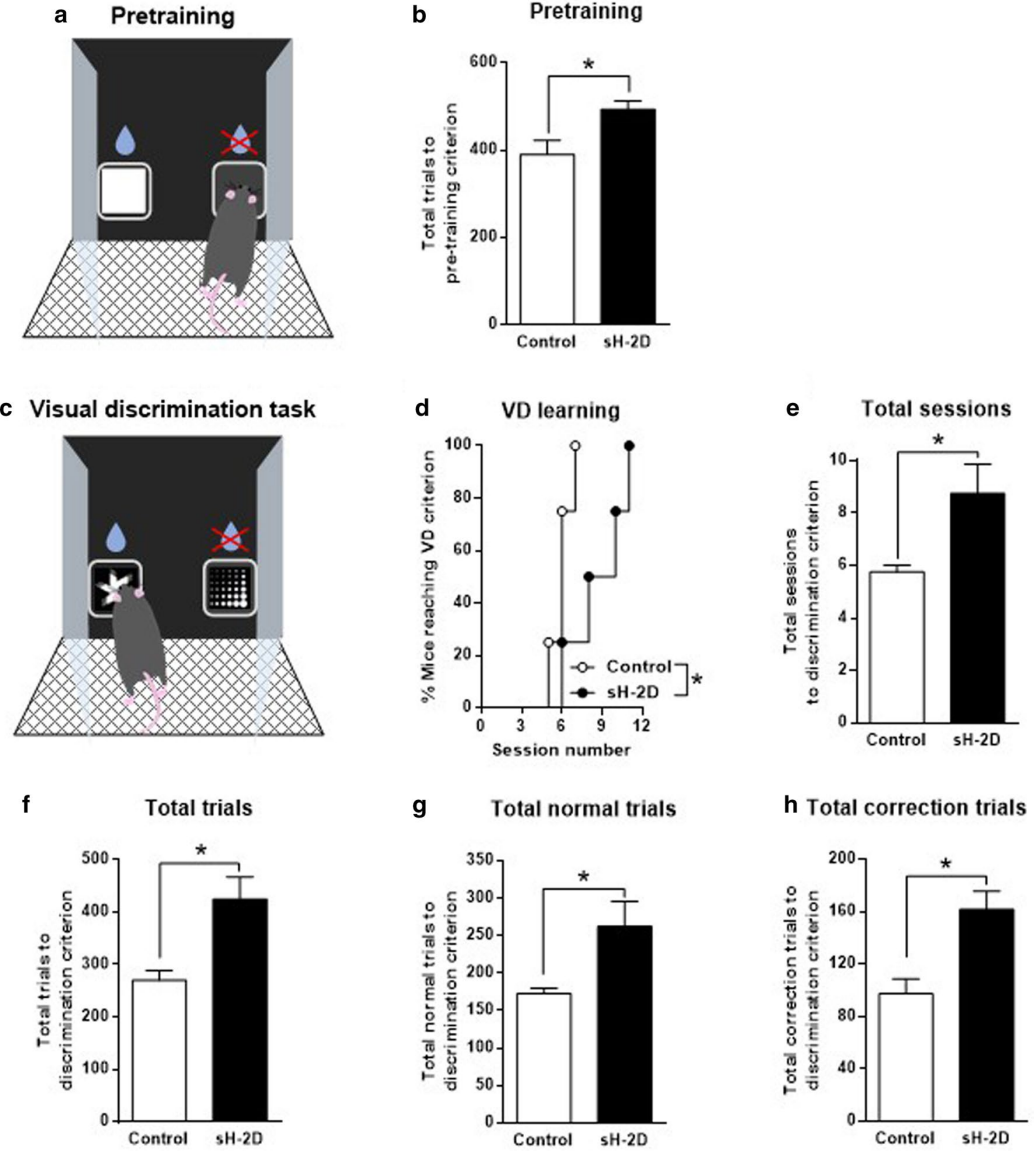
Performance of sH-2D-expressing mice in pretraining and VD task. **a** Introduction of incorrect response in the pretraining. **b** Total number of trials to reach the criterion in the pretraining session. **c** In the VD task, two stimuli (fan and marble) were presented simultaneously. The fan was shown as the correct response associated with a reward and the marble was an incorrect response with no reward. **d** VD learning. **e** Total number of sessions, **f** trials, **g** normal trials, and **h** correction trials to reach the discrimination criterion. Values indicate the mean ± SEM [control (n = 4 mice) and sH-2D (n = 4 mice)]

Mice were initially subjected to a 5-stages pretraining to gradually shape screen-touching behavior [18, 29]. Pretraining consisted of 5 stages (Fig. 1a). White plain and blank stimuli were used in the pretraining stages (Fig. 1b). In the pretraining, sH-2D-expressing mice required significantly more trials to reach the criterion (to reach 75% accuracy for at least 2 sessions) than control mice (t(6) = 2.74, P = 0.0338; Fig. 2b). Accordingly, sH-2D-expressing mice appear to have normal visuospatial and motor functions, but impaired reward-associated discriminative learning. The VD task was initiated when mice reached the criterion in pretraining. In this task, mice were required to touch a stimulus to obtain the liquid reward from a pair of stimuli (marble and fan; Fig. 1d, 2c). The VD learning was significantly slower in sH-2D-expressing mice than control mice (P = 0.0414; Fig. 2d). In fact, sH-2D-expressing mice needed more sessions (t(6) = 2.64, P = 0.0386; Fig. 2e), trials (t(6) = 3.35, P = 0.0154; Fig. 2f), normal trials (t(6) = 2.64, P = 0.0386; Fig. 2g), and correction trials (t(6) = 3.58, P = 0.0116; Fig. 2h) to reach the learning criteria (more than 80% accuracy on 2 consecutive days) than did control mice. Taken together, these results indicate that reward learning was significantly impaired by the overexpression of MHCI in astrocytes of the mPFC.

### Performance of sH-2D-expressing mice in reversal learning tasks

**Fig. 3:**
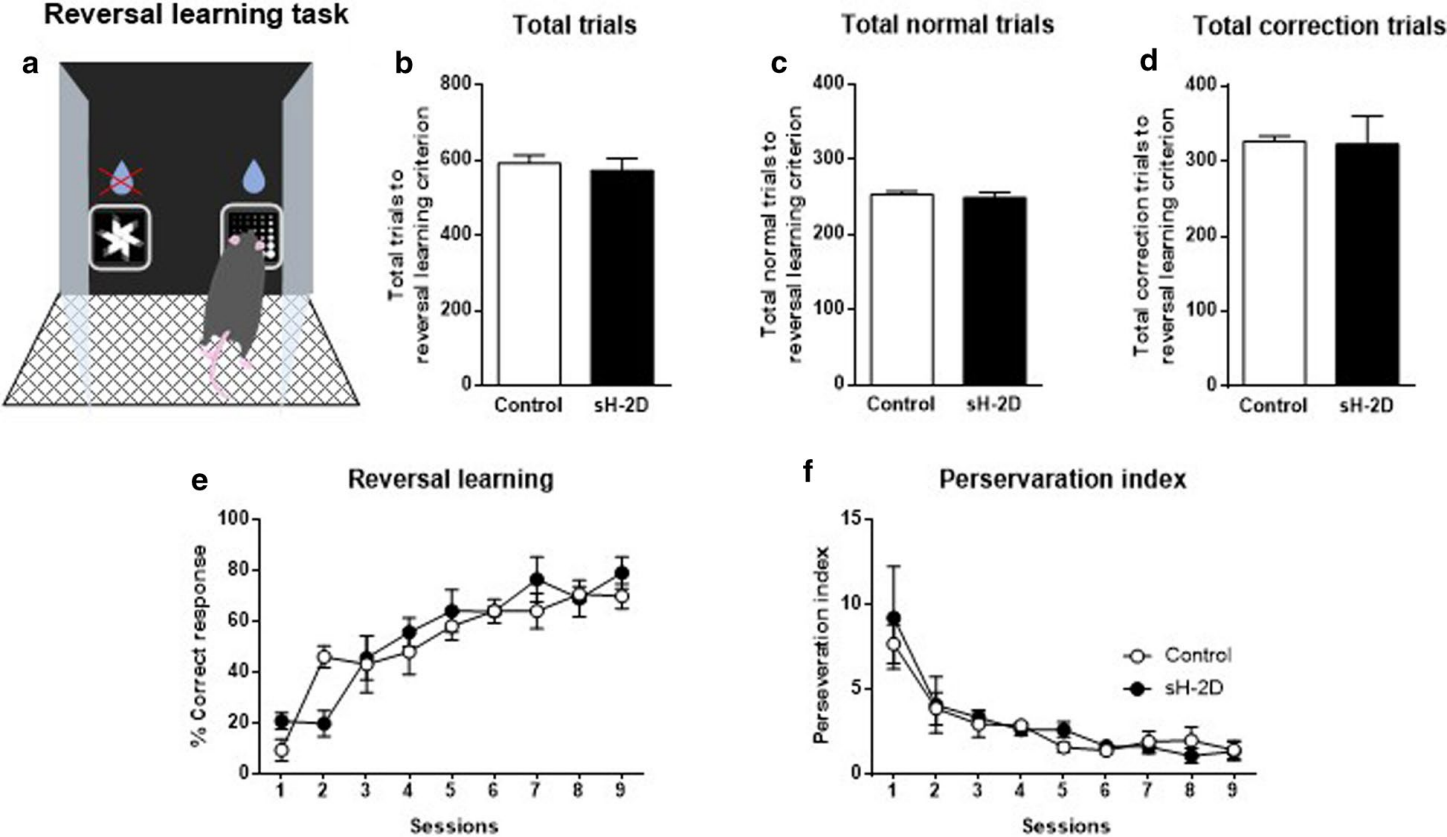
Performance of sH-2D-expressing mice in reversal learning tasks. **a** In reversal learning task, the contingency of the stimulus pair was reversed from the previous VD task. The previous reward stimulus (fan) became an incorrect response, while the previous non-rewarded stimulus (marble) became the correct response. **b** Total number of trials, **c** normal trials, and **d** correction trials to reach the reversal learning criterion. **e** Reversal learning. **f** The perseveration index. Values indicate the mean ± SEM [control (n = 4 mice) and sH-2D (n = 4 mice)]

In order to analyze the behavioral flexibility in sH-2D-expressing mice, the animals were then subjected to reversal learning in which the previously incorrect stimulus becomes the correct stimulus and vice versa (Fig. 3a). In a 9-day reversal learning task, the total trials (t(6) = 0.49, P = 0.6379; Fig. 3b), total normal trials (t(6) = 0.47, P = 0.6427; Fig. 3c) and total correction trials (t(6) = 0.09, P = 0.9291; Fig. 3d), were comparable between the groups. No significant differences were observed in the percentage of correct responses (group, F(1, 3) = 0.24, P = 0.6565; sessions, F(8, 24) = 38.88, P < 0.01; group × sessions, F(8, 24) = 1.80, P = 0.1272; Fig. 3e) or the perseveration index (group, F(1, 3) = 0.7183, P = 0.71; sessions, F(8, 24) = 9.14, P < 0.0001; group × sessions, F(8, 24) = 0.40, P = 0.9096; Fig. 3f) between the two groups of mice. Accordingly, the normal capability for reversal learning in sH-2D-expressing mice indicates that behavioral flexibility was minimally affected by the overexpression of astroglial MHCI in the mPFC.

### Morphology of DMS and DLS neurons in sH-2D-expressing mice

**Fig. 4:**
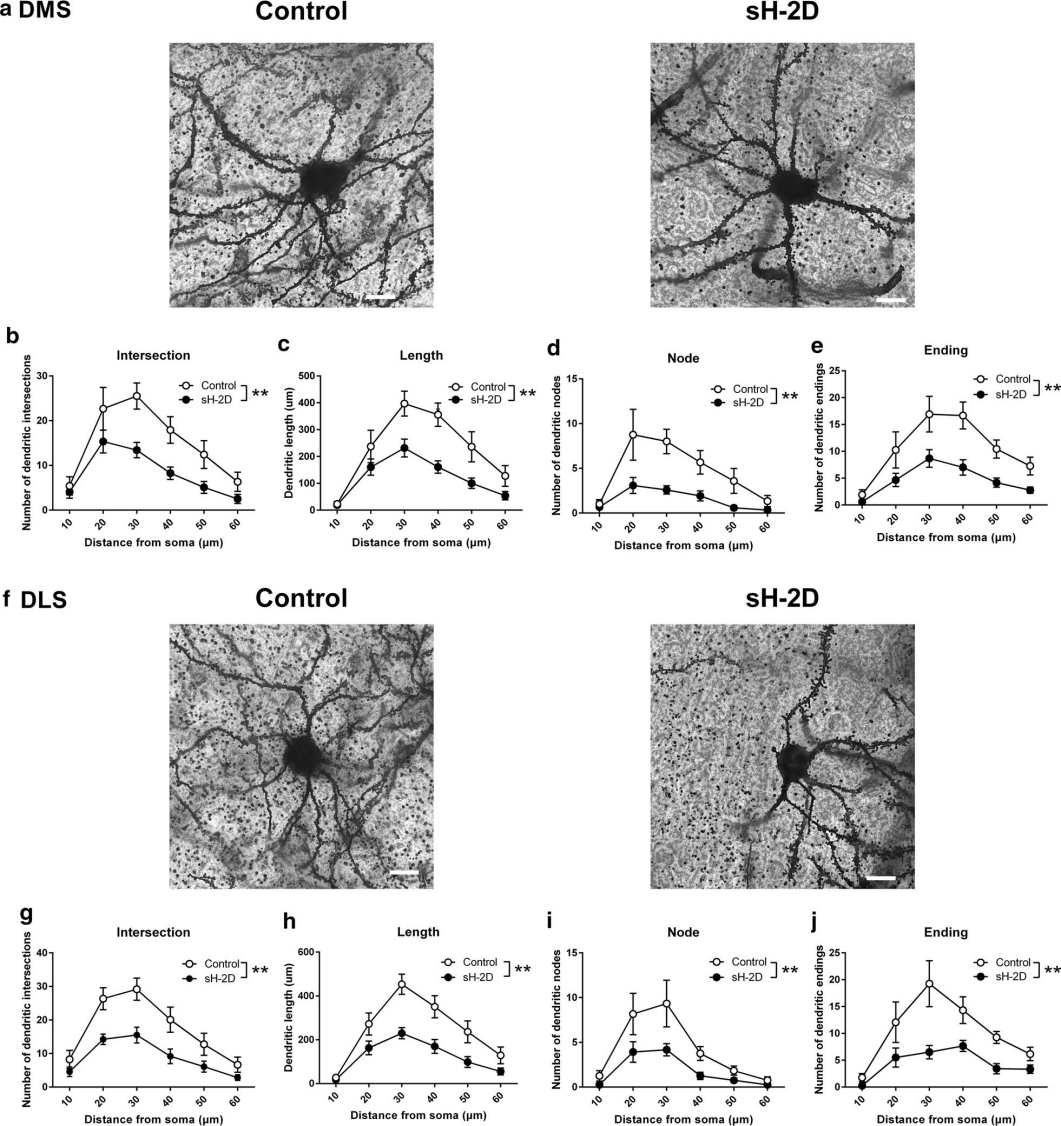
Morphology of DMS and DLS neurons in sH-2D-expressing mice. **a** Representative images show DMS neurons in control and sH-2D-expressing mice. **b** Quantification of the intersection, **c** length, **d** node, and **e** ending of DMS neurons in control and sH-2D-expressing mice. **f** Representative images showing DLS neurons in control and sH-2D-expressing mice. **g** Quantification of the intersection, **h** length, **i** node, and **j** ending of DMS neurons in control and sH-2D-expressing mice. Scale bar indicates 10 μm. **p < 0.01 significantly different from control mice. DMS, dorsomedial striatum; DLS, dorsolateral striatum. Values indicate the mean ± SEM [control (n = 12 neurons from 4 mice) and sH-2D (n = 12 neurons from 4 mice)]

The dorsal striatum is anatomically divided into DMS and DLS. These areas are involved in VD learning [18, 21]. These striatal regions receive excitatory inputs from two major sources, the cortex and thalamus, which control contextual, motor, and perceptual decisions [24, 30]. Therefore, we analyzed the morphology of DMS and DLS neurons in control and sH-2D-expressing mice by the Sholl analysis (Fig. 4a, f). The dendrites of DMS neurons in sH-2D-expressing mice showed significantly fewer intersections (group, F(1, 22) = 27.79, P < 0.0001; intersection, F(5, 110) = 14.04, P < 0.0001; group × intersection interaction, F(5, 110) = 1.173, P = 0.3271; Fig. 4b), shorter lengths (group, F(1, 22) = 28.70, P < 0.0001; length, F(5, 110) = 17.53, P < 0.0001; group × length, F(5, 110) = 1.870, P = 0.1053; Fig. 4c), and less nodes (group, F(1, 22) = 28.39, P < 0.0001; node, F(5, 110) = 7.018, P < 0.0001; group × node, F(5, 110) = 1.746, P = 0.1301; Fig. 4d) and endings (group, F(1, 22) = 26.82, P < 0.0001; ending, F(5, 110) = 10.60, P < 0.0001; group × ending, F(5, 110) = 1.23; P = 0.3000; Fig. 4e) per Sholl segment, particularly those 10–60 μm from the soma, than those in control mice. Similarly, the dendrites of DLS neurons in sH-2D-expressing mice showed significantly fewer intersections (group, F(1, 22) = 26.71, P < 0.0001; intersection, F(5, 110) = 17.57, P < 0.0001; group × intersection, F(5, 110) = 1.543, P = 0.1824; Fig. 4g), shorter lengths (group, F(1, 22) = 32.42, P < 0.0001; length, F(5, 110) = 22.38, < 0.0001; group × length, F(5, 110) = 2.457, P = 0.0376; Fig. 4h), and less nodes (group, F(1, 22) = 9.79, P < 0.0001; node, F(5, 110) = 12.63, P < 0.01; group × node, F(5, 110) = 1.54, P = 0.1844; Fig. 4i) and endings (group, F(1, 22) = 15.99, P = 0.0006; ending, F(5, 110) = 10.48, < 0.0001; group × ending, F(5, 110) = 2.14, P = 0.0664; Fig. 4j) per Sholl segment than those in control mice. These results suggest that overexpression of astroglial MHCI in the mPFC decreases the dendritic complexity in the striatum.

### Dendritic spine formation in the DMS and DLS of sH-2D-expressing mice

**Fig. 5:**
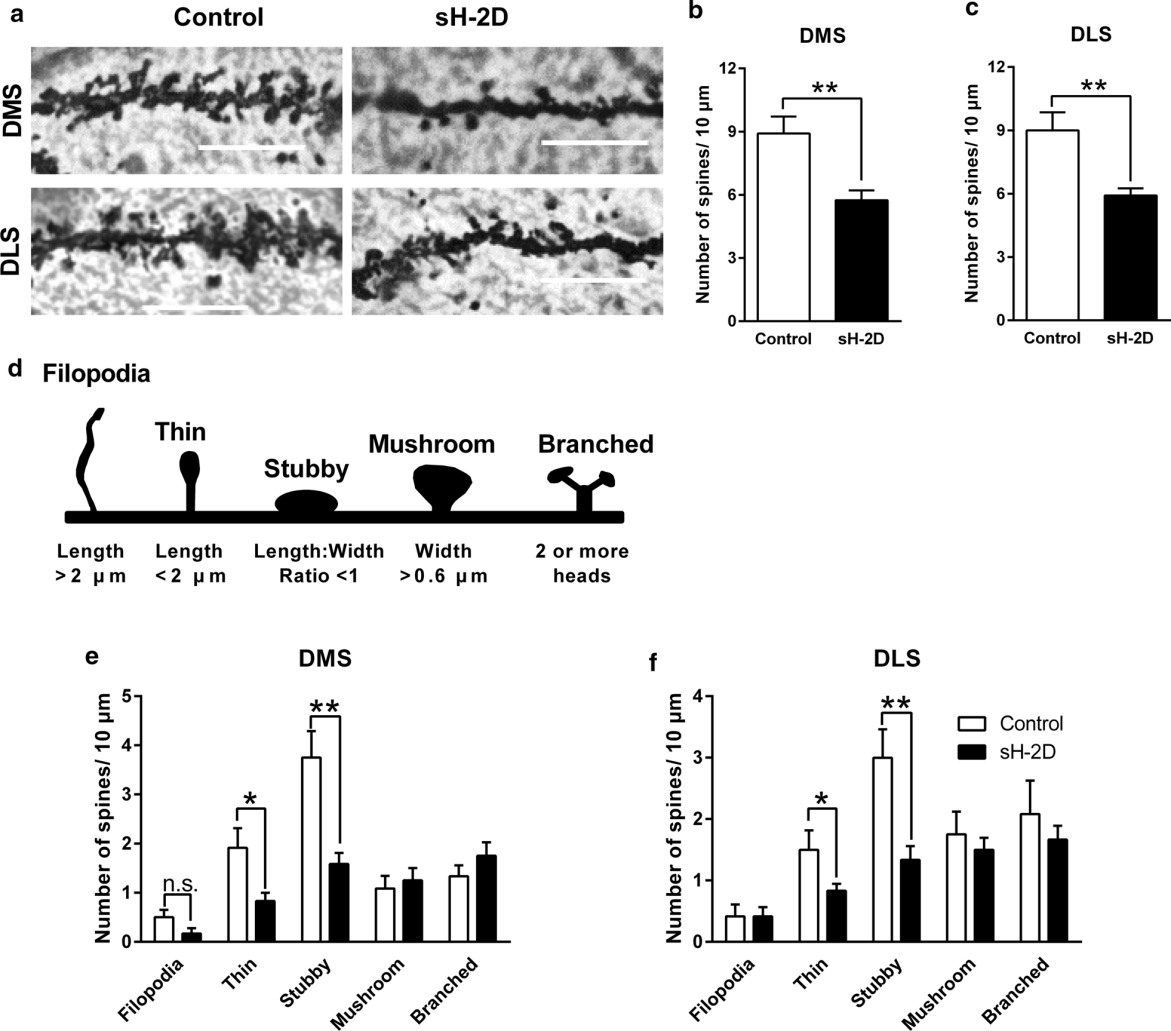
Dendritic spine formation in the DMS and DLS in sH-2D-expressing mice. **a** Representative images show DMS and DLS spines in control and sH-2D-expressing mice. Quantification of the spines in the **b** DMS and **c** DLS. **d** Morphological classification of dendritic spines. Quantification of the filopodia, thin, stubby, mushroom and branched spines in the **e** DMS and **f** DLS. *p < 0.05; **p < 0.01 significantly different from control mice. DMS, dorsomedial striatum; DLS, dorsolateral striatum. Scale bar indicates 10 μm. Values indicate the mean ± SEM [control (n = 12 dendrites from 4 mice) and sH-2D (n = 12 dendrites from 4 mice)]

Because the dendritic complexity is decreased in the striatum of sH-2D-expressing mice, we hypothesized the overexpression of astroglial MHCI in the mPFC may disrupted the spine maturation process. Therefore, we further analyzed the density of spine and its subtypes (Fig. 5a). The number of spines was significantly decreased in the DMS (t(22) = 3.42, P = 0.0025; Fig. 5b) and DLS (t(22) = 3.64, P = 0.0028; Fig. 5c) of sH-2D-expressing mice as compared to those in control mice. Figure 5d shows the criterion to determine the spine subtypes. The thin and stubby types of spine were significantly decreased in both DMS (thin, t(22) = 2.51, P = 0.0199; stubby, t(22) = 3.70, P = 0.0012; Fig. 5e) and DLS (thin, t(22) = 2.42, P = 0.0238; stubby, t(22) = 3.25, P = 0.0037; Fig. 5f). These results suggest that overexpression of astroglial MHCI in the mPFC decreases the thin and stubby types of spine in the striatum.

## Discussion

Touchscreen-based behavioral assays are parallel with computerized tasks used in human patients [16, 31]. Mice carrying human disease-related genetic mutations exhibit cognitive impairments in the touchscreen-based VD task [17–20, 32]. Previous studies suggested that normal performance in the VD task depends on the intact function of the corticostriatal circuit [18, 21], which consists of the PFC, striatum, and thalamus, and is considered to be important for learning behaviors in humans, primates, and rodents [33–35]. Therefore, the function of the mPFC was tested using the VD task, which relies on this area and its projection terminals on the computation of cognitive outputs with high translational validity. The first novel result of the present study is regional and cell specificities in the role of astroglial MHCI in the touchscreen-based VD task. The animal model used here allowed astrocytes to be specifically targeted within the mPFC, without affecting other types of cells, by using the AAV under the control of the GfaABC1D promotor [15].

The task started with 5 stages of pretraining prior to the VD task. By gradually completing the 5 stages, mice learned how to operate the touchscreen to get a reward. The results obtained showed that the performance of sH-2D-expressing mice was normal in the response phase sessions (stages 1–4), but impaired in the punish phase session (stage 5). Therefore, sH-2D-expressing mice appear to have normal visuospatial and motor functions, but impaired reward-associated discriminative learning. Similar to stage 5, the VD task requires to learn that one of two stimuli (marble and fan) simultaneously displayed on the screen is associated with the reward. The total numbers of sessions, trials, normal trials, and correction trials were significantly higher in sH-2D-expressing mice than in control mice, indicating that reward learning was significantly impaired by the overexpression of MHCI in astrocytes in the mPFC. However, no significant differences were observed between sH-2D-expressing mice and control mice in the performance of reversal learning, in which the previously incorrect stimulus becomes the correct stimulus and vice versa. The perseveration index, a paradigm that is often used in reversal learning to evaluate behavioral flexibility in mice, was also similar between two groups of mice. Previous studies have indicated that activation of astrocytes in the mPFC impairs attention and reversal learning functions [36, 37], and lesions in the mPFC have a negative impact on the performance of reversal learning [38]. These previous findings suggest a critical role for the mPFC in reversal learning. The normal capability for reversal learning in sH-2D-expressing mice indicates that behavioral flexibility was minimally affected by the overexpression of astroglial MHCI in the mPFC. One might concern the type I error because the number of mice used in the touchscreen-based tasks is quite low. We cannot exclude the possibility, but it should be noted that this operant behavioral test is an automated one with high reproducibility and low variability, which is more sensitive to detect cognitive abnormality in mice than the other respondent behavioral tests such as the novel object recognition test [16–18, 39].

Astrocytes play critical roles in CNS homeostasis by supporting neuronal metabolism and excitability, structuring the blood–brain-barrier, and limiting the synapse microenvironment [40]. They provide neurotrophic support, promote synapse formation and plasticity, and regulate synaptic transmission by interacting with dendritic spines and neuronal cell bodies [41–43]. Combining our previous findings [15] with the VD deficit observed here, it is suggested that the astroglial MHCI may affect neighboring cells and lead to a reduction in dendritic spine density with microglial activation and then decrease in parvalbumin-positive interneurons. These morphological changes may result in dysfunctions in the mPFC associated with cognitive function including novel object recognition and VD learning [15, 44].

Corticostriatal projections are massive and broad and arise from all cortical regions [45, 46]. Pyramidal neurons in the mPFC provide cortical input, and their axons terminate primarily on the spines of striatal medium spiny neurons. These medium spiny neurons represent more than 90% of the striatal neuronal population in rodents [45]. Instrumental action and outcome behaviors depend on the striatum and its connections with the mPFC [22, 23]. Neuronal manipulations or lesion studies demonstrated that the dorsal striatum is associated with VD learning [18, 21]. Cortical synaptic inputs into the striatum are important for the maturation of the dendritic arborization of striatal medium spine neurons [47]. The present study has raised an important question if the neuronal changes could be observed only in the areas directly linked to the mPFC.

We have previously demonstrated that treatment with GW4869 which impairs exosome synthesis significantly ameliorates the behavioral and neuropathological changes in sH-2D-expressing mice [15]. Accordingly, the overexpression of MHCI in astrocytes of mPFC induces microglial proliferation and the decrease in neuronal numbers of the mPFC, and affects dendritic complexity and spine density in the striatum, specifically the lowered number of spinal thin and stubby types. It is possible that all of these could be triggered by the exosomes that are secreted by MHCI-expressing astrocytes in the mPFC. The manipulation in the mPFC may have delayed the spine maturation thereafter leading to the decreased dendritic complexity in the striatum. More direct evidence is needed to test this hypothesis in future studies.

We previously demonstrated that treatment with polyinosinic-polycytidylic acid (poly:C) in adult mice significantly increased MCHI, interferon, tumor necrosis factor-a, and interleukin-6 mRNA expression levels in the mPFC [15]. Under pathological conditions, such as viral infection, astrocytes secrete several inflammatory cytokines and chemokines that interrupt local immune responses, which may contribute to the expansion of primary lesions, leading to further neuronal loss [48, 49]. Activated microglia induce neuronal degeneration or death, both of which are associated with mental disorders such as schizophrenia and depression [50–52]. Overexpression of astroglial MHCI in the mPFC increases the number of microglia and the glutamatergic transporter expression (e.g. glutamate transporter 1) [15, 53]. We speculate the expression of striatal microglia may have altered by the glutamatergic projection from mPFC. As mentioned above, the inflammatory cytokines and chemokines may contribute to these processes, but future studies are necessary to prove it.

In conclusion, the present results suggest that the overexpression of MHCI in astrocytes in the mPFC results in impaired VD learning, which is associated with decreased dendritic complexity and spine density in medium spine neurons in the dorsal striatum and mPFC.

## Data Availability

All data used in this study are available from the corresponding author on reasonable request.

## Abbreviations

AAV: Adeno-associated virus
CNS: Central nervous system
DLS: Dorsolateral striatum
DMS: Dorsomedial striatum
GFAP: Glial fibrillary acidic protein
ITI: Inter-trial interval
MHC: Major histocompatibility complex
MHCI: MHC class I
PCR: Polymerase chain reaction
VD: Visual discrimination
mPFC: Medial prefrontal cortex
poly:C: Polyinosinic-polycytidylic acid
sH-2D: Soluble form of H-2D
sH-2D-expressing mice: Mice expressing the soluble form of H-2D in the mPFC
